# Prostate specific antigen testing policy worldwide varies greatly and seems not to be in accordance with guidelines: a systematic review

**DOI:** 10.1186/1471-2296-13-100

**Published:** 2012-10-11

**Authors:** Saskia Van der Meer, Sabine AM Löwik, Willem H Hirdes, Rien M Nijman, Klaas Van der Meer, Josette EHM Hoekstra-Weebers, Marco H Blanker

**Affiliations:** 1 Isala clinics, Department of Urology, Groot Wezenland 20, 8011, JW, Zwolle, The Netherlands; 2 Department of General Practice, University of Groningen, University Medical Center Groningen, Hanzeplein 1, P.O.Box 30.001, 9700, RB, Groningen, The Netherlands; 3 Department of Urology, University of Groningen, University Medical Center Groningen, Hanzeplein 1, P.O.Box 30.001, 9700, RB, Groningen, The Netherlands; 4 University of Groningen, University Medical Center Groningen, Psychosocial services Hanzeplein 1, P.O.Box 30.001, 9700, RB, Groningen, The Netherlands

**Keywords:** Prostate specific antigen, PSA, Follow-up, General practitioners, Non-urologic hospitalists, Guidelines, Systematic review

## Abstract

**Background:**

Prostate specific antigen (PSA) testing is widely used, but guidelines on follow-up are unclear.

**Methods:**

We performed a systematic review of the literature to determine follow-up policy after PSA testing by general practitioners (GPs) and non-urologic hospitalists, the use of a cut-off value for this policy, the reasons for repeating a PSA test after an initial normal result, the existence of a general cut-off value below which a PSA result is considered normal, and the time frame for repeating a test.

*Data sources*. MEDLINE, Embase, PsychInfo and the Cochrane library from January 1950 until May 2011.

*Study eligibility criteria*. Studies describing follow-up policy by GPs or non-urologic hospitalists after a primary PSA test, excluding urologists and patients with prostate cancer. Studies written in Dutch, English, French, German, Italian or Spanish were included. Excluded were studies describing follow-up policy by urologists and follow-up of patients with prostate cancer. The quality of each study was structurally assessed.

**Results:**

Fifteen articles met the inclusion criteria. Three studies were of high quality. Follow-up differed greatly both after a normal and an abnormal PSA test result. Only one study described the reasons for not performing follow-up after an abnormal PSA result.

**Conclusions:**

Based on the available literature, we cannot adequately assess physicians’ follow-up policy after a primary PSA test. Follow-up after a normal or raised PSA test by GPs and non-urologic hospitalists seems to a large extent not in accordance with the guidelines.

## Background

Recently, Vedel *et al*. showed that although most guidelines are cautious about screening for prostate cancer, using PSA tests is routine practice in a variety of healthcare systems, both in North America and Europe [1].

The effect of prostate cancer screening on mortality and quality of life remains unclear [2]. Even after the European Randomised Study of Screening for Prostate Cancer (ERSPC)- the only study that showed a clear positive effect on prostate specific mortality - it is unknown which patients may benefit from screen-detected early diagnosis [3]. Still, Prostate Specific Antigen (PSA) tests are used regularly in patients with and without lower urinary tract symptoms (LUTS) [4-6].

The PSA test has several limitations: Due to its low specificity, an elevated PSA level does not necessarily indicate the presence of prostate cancer, as it can also be caused by benign prostate hyperplasia (BPH), urinary retention or prostatitis. Furthermore, test sensitivity at the applied normal values is suboptimal: a normal PSA value does not rule out prostate cancer [7,8]. Also, an abnormal PSA test result can have a great impact on a patients’ mental health [9].

Because of these difficulties, guidelines on the use of the PSA test have been developed. The American Urological Association (AUA) PSA Best Practice Statement Update 2009 recommends to start regular screening for prostate cancer at the age of 40 years in patients with an anticipated lifespan of more than 10 years [9]. They no longer mention a threshold value of PSA to prompt follow-up, but advise basing the decision primarily on PSA and Digital Rectal Examination (DRE) results. Also, free and total PSA, patient’s age, PSA velocity, PSA density, family history, ethnicity, prior biopsy history and comorbidities need to be taken into account [9]. The European Association of Urology (EAU) recommends follow-up after an abnormal DRE or elevated serum PSA measurement, with a cut-off level of <2.5-3 ng/ml for younger men [10]. The European Society for Medical Oncology does not recommend screening for prostate cancer. However, they do advise prostate biopsies after an abnormal DRE or elevated PSA values, although no PSA cut-off value is mentioned [11].

It is unclear whether (and how) physicians use these guidelines and what cut-off values are being considered in daily practice. Especially follow-up by non-urologists is not clearly described in the literature. We conducted a systematic review of the literature on how follow-up after PSA testing is being conducted. We focused on the following questions. What is the policy of general practitioners (GPs) and non-urologic hospitalists after an abnormal PSA test result and can a cut-off value be determined for this policy? What are the reasons for repeating a PSA test after an initial normal result, what is the cut-off value used for a normal PSA test result and what is the time frame for repeating the test?

## Methods

### Identification and selection of the literature

In November 2009, a search of the literature was performed in duplicate (SM and SAML) to identify relevant publications on follow-up by GPs and non-urologic hospitalists after a PSA test. We used MEDLINE, Embase, PsycInfo and the Cochrane library to search for articles published from 1950 until October 2009. This search was updated in May 2011. The terms general practitioner and non-urologic hospitalist were linked by the Boolean operator OR. The Boolean operator AND linked these terms to the term PSA and to the terms follow-up or referral or consultation. For all these keywords one or more synonyms were used and all items were searched using “All fields” (Additional file 1: Appendix 1).

We included studies if the following criteria were met: study contained original data, it described actions undertaken by GPs or non-urologic specialists following an initial PSA test (no action, repeat the test, or referral to a urologist), and the article was written in Dutch, English, French, German, Italian or Spanish. Excluded were studies describing actions undertaken by urologists and studies on patients with prostate cancer. Titles and abstracts of the identified studies were checked and the full text of these publications were read to find out whether inclusion criteria were met. Also, we screened reference lists of all relevant articles for other relevant studies. We contacted the authors if an article did not provide enough information to adequately assess inclusion criteria.

The main characteristics of the included publications were extracted by two authors (SM and SAML) using standardized forms.

### Quality assessment

A difference in validity of included studies may affect the conclusions of a systematic review. Therefore, we assessed the quality of each study: two reviewers (SM and SAML) independently scored the quality of the included studies using a set of criteria. These criteria were a combination of the quality criteria described by Harden *et al*. and Prins *et al*. [12,13]. Each item was scored a “0” if the criterion was not met or if it was unclear whether the criterion was fulfilled, a “1” was given when that particular criterion was fulfilled. The sum of these scores indicated the quality score for each study. Disagreement was resolved by consensus or by a third reviewer (WHH) in case of persisting disagreement. The preferred study design was a prospective cohort study. In our study, 10 quality items and 5 informativity items were scored (Additional file 1: Appendix 2). The informativity score was not included in the quality score, as this reflects the quality of the manuscript rather than the quality of the study. Studies receiving more than 75% of the quality points (more than 7 points) were considered studies of high quality. The quality of database studies was assessed using the same scoring system, but three items of this system did not apply to this type of research, thus restricting the total score of these studies to 7 points and therefore defining a high quality database study as a study with more than 5 points.

### Data analysis

Most studies in this review were too heterogeneous in their study design to apply statistical analysis of the data. We compared the results of the studies included using the quality score and study design (survey study or database study).

## Results

### Selection and quality of studies

**Figure 1:**
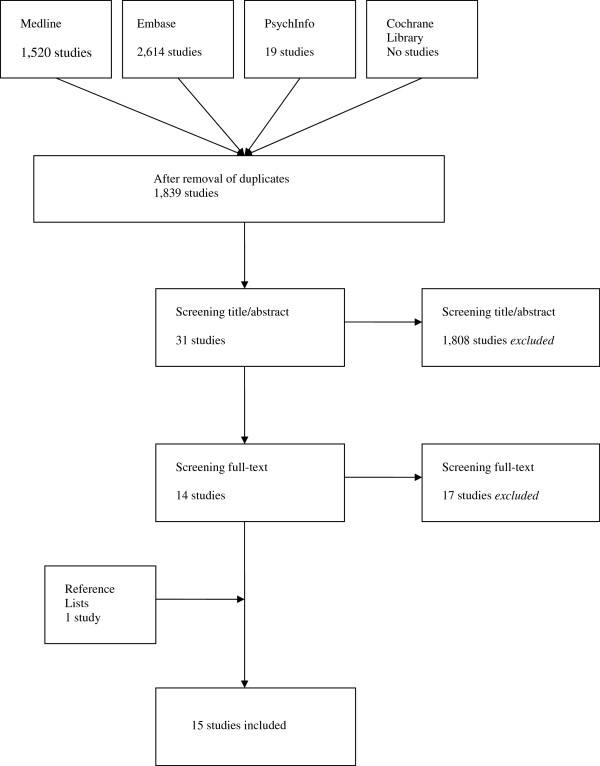
Systematic review flow diagram.

No search results were found in the Cochrane Library. Embase identified a total of 2,614 titles. From MEDLINE 1,520 titles were extracted. PsychInfo revealed 19 articles. After reviewing title and abstract 31 articles were read fulltext. Fourteen articles met our inclusion criteria. Screening the reference list of these articles revealed one additional study meeting the inclusion criteria (Figure 1) [6,7,14-26].

Study characteristics of the 15 included studies are presented in Table 1. Three studies were database related, one study combined questionnaires with database research, and 11 studies were questionnaire-based. One study comprised of an abstract only.

**Table 1:** Characteristics of included studies

**Reference number**	**Year**	**Country**	**Design**	**Participants**	**Source population and response rate**	**QS**
[14]	2006	USA	Database	GPs & internists	505 patients aged 75 years or older, not previously diagnosed with prostate cancer who underwent a PSA test between 1998 and 2004	4
[24]	2000	Spain	Database	GP	94 patients not previously diagnosed with prostate cancer with a PSA ≥ 4 in 1998, selected from the pertinent laboratory	6
[21]	1998	USA	Database	Internists	1,448 patients not previously diagnosed with prostate cancer who in 1993 underwent a PSA test	5
[23]	2008	UK	Database & survey	GPs	Database: 709 patients pre-guideline launch and 1,040 men post-guideline launch with PSA ≥ 3 ng/ml and ≥ 1 PSA test by GPs	7 (survey)
					515 patients pre-guideline launch and 607 post-guideline launch with normal test-result (< 3) (random 25% sample of normal PSA tests by GP)	
					Questionnaire: 69 GPs from England and Wales, registered for PSA testing with the National External Quality Assessment Service, 48 responded, response rate 70%	
[18]	2008	France	Survey	GPs	All 1,339 GPs in Auvergne (France) registered with the Urssaf in 2006, 658 responded, response rate 49,1%	5
[26]	2008	UK	Survey	GPs	502 PCPs in West Suffolk and Ipswich Hospital area (UK), 192 responded, response rate 38%	6
[7]	2005	Denmark	Survey	GPs	325 GPs in Northern Denmark (23% woman), 291 responded, response rate 90%	8
[6]	2003	Ireland	Survey	GPs	400 GPs from Northern Ireland randomly selected by computer, 282 responded, response rate 71%	6
[15]*	2002	UK	Survey	GPs	200 GPs registered in the East Surrey region, 118 responded, response rate 59%	2
[19]	1995	USA	Survey	GPs	All 149 OAFP GPs from the Oklahoma City area and 151 randomly selected OAFP physicians outside this area, 152 responded, response rate 53%	9
[22]	1995	UK	Survey	GPs/general surgeons/ geriatricians/FHSA	500 GPs, associated with the Western General Infirmary in Edinburgh, Scotland, 118 responded, response rate 23,6%	4
					373 general surgeons, 85 responded, response rate 22,8%	
					712 geriatricians, 160 responded, response rate 22,5%	
					115 FHSAs, 58 responded, response rate 50,4% (and 320 urologists, results excluded from this SR)	
[17]	2007	Japan	Survey	Internists/general surgeons (PCP)	935 PCPs (internist and general surgeons not in hospital and no urologist) in South metropolitan Tokyo, 281 responded, mean response rate 30% (varying per question)	5
[16]	1998	USA	Survey	GPs/internists (PCP)	All physicians from the AMARP, listing themselves in general internal medicine, family medicine (or urology, results excluded in this SR). 444 PCPs responded, response rate 51%	6
[20]	1996	USA	Survey	GPs/internists (PCP)	1.816 PCPs randomly selected from all Arizona-licensed physicians from the BMESA who selfreported to be GP, family practitioner or internist. 68 were subsequently excluded (various reasons) and 57 were unreachable. Overall, 141 eligible physicians completed the survey, response rate 42,9%	10
[25]	1996	USA	Survey	Internists/medical subspecialists (PCP)	All PCPs in Brooklyn, New York with the MSSNY, 311 PCPs responded, response rate 28%, including 134 GPs (43,1%) and 177 internists/medical subspecialists (56,9%)	4

Table order according to study design (database or survey studies), participants (starting with GPs) and year of publication (starting with most recent publications)

Abbreviations: *QS* Quality Score; *PCP* Primary Care Physician; *GP* General Practitioner; *OAFP* Oklahoma Academy of Family Physicians; *SR* Systematic Review; *FHSA* Family Health Service Advisor; *AMARP* American Medical Association Registry of Physicians; *BMESA* Board of Medical Examiners of the State of Arizona; *MSSNY* Medical Society of the State of New York.

* abstract only.

The study population of the questionnaire studies varied between 48 and 658 physicians. The response rate varied from 28% to 90%. The database studies had study populations varying from 94 to 1,647 patients. One database study only included patients of 75 years or older.

Quality scores are presented in Tables 1 and 2. Three survey studies and one database study were of high quality. On average, 2.8 items (range 0–5) on external validity were scored positively as were 2.8 items (range 1–5) on internal validity.

**Table 2:** Quality scores of included studies

**Reference number**	**Year**	**External validity**						**Internal validity**						**Informativity**						**Disagree**
		**a**	**b**	**c**	**d**	**e**	**sum**	**f**	**g**	**h**	**i**	**j**	**sum**	**k**	**l**	**m**	**n**	**o**	**sum**	
*Questionnaire studies*																				
[23]	2008	0	1	1	0	1	3	1	0	1	1	1	4	1	1	1	1	1	5	**d,f,h**
[18]	2008	0	0	0	1	1	2	1	0	1	1	0	3	1	1	1	1	1	5	**g**
[26]	2008	1	1	0	1	0	3	1	0	0	1	1	3	1	1	1	0	0	3	**n**
[17]	2007	1	1	0	0	0	2	1	1	0	1	0	3	1	1	1	0	0	3	**d,n**
[7]	2005	1	1	1	1	0	4	1	1	0	1	1	4	1	1	1	1	1	5	
[6]	2003	0	1	1	1	0	3	1	0	0	1	1	3	0	1	1	1	1	4	
[15]^i^	2002	0	0	0	0	0	0	1	0	0	0	1	2	0	1	1	0	0	2	**d,k**
[16]	1998	1	1	0	1	0	3	1	1	0	1	0	3	1	1	1	1	1	5	
[20]	1996	1	1	1	1	1	5	1	1	1	1	1	5	0	1	1	1	1	4	**i**
[25]	1996	0	1	0	0	0	1	1	1	0	1	0	3	0	1	1	1	1	4	**d**
[19]	1995	1	1	1	1	1	5	1	0	1	1	1	4	0	1	1	1	1	4	**l,o**
[22]	1995	0	0	0	1	0	1	1	0	0	1	1	3	1	1	0	1	1	4	**i,m**
*Database studies*^ii^																				
[14]	2006	1	1	**-**	0	1	3	0	**-**	**-**	1	0	1	1	1	1	1	1	5	**d**
[24]	2000	1	1	**-**	1	1	4	0	**-**	**-**	1	1	2	0	1	1	1	1	4	
[21]	1998	1	1	**-**	0	1	3	0	**-**	**-**	1	1	2	1	1	1	1	1	5	

^i^ Abstract only.

^ii^ Categories c, g and h did not apply to database studies (−).

### Referral rate after normal PSA test

As most studies defined a PSA < 4 ng/ml as normal, this value was chosen to differentiate between a normal and abnormal PSA test result (Tables 3 and 4). After a normal PSA test result (PSA < 4, or PSA ≤ 4, or age-specific cut off values, Table 3) referral was described in 2 studies [14,23], and varied from 0% reported by GPs to 28.6% referred by GPs and internists [14,23].

**Table 3:** Follow-up after normal PSA values

**Reference number**	**Country**	**Cut off (ng/ml)**	**Referral (%)**	**Repeat test (%)**	**No action (%)**
*Database studies*					
[14]	USA	PSA < 4	28.6		71
[21]	USA	PSA < 4		37.7	
*Survey studies*					
[23]	UK	PSA < 3	0	1.3	98.7
		PSA 0.1-4	7		
[18]	France	NS		54.3	27.5
[22]	UK	PSA ≤ 4		86.9	
		GPs		82.8	
		General surgeons		64.3	
		Geriatricians		89.4	
		FHSA			
[17]	Japan	PSA ≤ 4		17	

Abbreviations: *NS* Not Specified; *GP* General Practitioner; *FHSA* Family Health Service Advisor.

**Table 4:** Follow-up after abnormal PSA values

**Reference number**	**Country**	**PSA Cut off (ng/ml)**	**Referral (%)**	**Repeat test (%)**	**No action (%)**	**PSA > 10 referral (%)**
*Database studies*						
[14]	USA	PSA ≥ 4	51.9		48	
		Age-specific cut off:	58		42	
		6.5 (≥ 75 yr)				
[24]	Spain	PSA ≥ 4	31		69	
[21]	USA	PSA ≥ 4	86	56.1		100
*Survey studies*						
[23]	UK	PSA ≥ 3	18.2	15.7	67	82.7
		PSA ≥ 4	30			
		Mean (calculated)	28.6			71.4
		50–59 yr: 6.2				
		70–84 yr: 13				
[18]	France	NS	10.5			
[26]	UK	Median (calculated)				
		45 yr: 4.5				
		55 yr: 5.5				
		65 yr: 6.5				
		75 yr: 6.5				
		85 yr: 7.5				
[7]	Denmark	mean 5 (calculated)				
[6]	Ireland	median 5.4 (calculated)		55		
[15]*	UK	NS				28
[19]	USA	NS	33			93
[22]	UK	PSA > 4	85.8			
		GPs	78.4			
		General surgeons	85.3			
		Geriatricians	100			
		FHSA				
[17]	Japan	PSA > 4	73			20
[16]	USA	PSA 4-10	Age specific:			
			50–59 years: 69			
			60–69 years: 67			
			70–74 years: 58			
			75–79 years: 52			
			≥ 80 years: 45			
[20]	USA	NS	Most doctors refer with PSA 4-10			13
[25]	USA	PSA > 4	67.8			

Abbreviations: *yr* year; *NS* Not Specified; *GP* General Practitioner; *FHSA* Family Health Service Advisor.

* abstract only.

### Referral rate after moderately increased PSA (≥ 4 ng/ml)

Three database studies showed a referral rate of 31 to 86% after a PSA ≥ 4 ng/ml (Table 4). More patients were referred in the USA [14,16,19,21,25] than in Spain [24], and internists [21] referred more often than GPs [19,22-24]. Seven survey studies described referral in 10.5 to 100% of the PSA values > 4 ng/ml (Table 4). GPs mentioned referring more often compared to primary care practitioners (PCPs) [18-20,22-25]. Non-urologic hospitalists mentioned referring equally often as PCPs [16,17,21,22,25]. One study reported high overall referral rates for GPs (85.5%) and non-urologic hospitalists (79-100%) compared to the other studies [22]. This was also the only study describing high percentage of follow-up for non-urologic hospitalists.

### Referral rate after clearly increased PSA (> 10 ng/ml)

One survey reported that 93% of the physicians (GPs) referred at PSA > 10 ng/ml [19]. However, three other surveys mentioned that only 13%, 28% and 20% of the physicians (GPs/PCPs) respectively referred at PSA > 10 ng/ml [15,17,20].

Reported referral rates in the USA were 33% in 1995 (GPs), 60% and 86% in 1998 (PCPs, internists and GPs respectively, Table 3) [16,19], and 52% in 2006 (GPs) [14,21]. In the UK, referral rates were 86% and 30% in 1995 and 2008, respectively (all GPs) [22,23]. Cut off values for referral were reported in 4 studies and varied between a mean of 5 ng/ml to 13 ng/ml for patients aged 70–84 years old (Table 4) [6,7,23,26].

### Repeat testing after normal PSA (< 4 ng/ml)

Repeat testing varied greatly also. One UK survey showed a high repeat rate reported by all physicians [22], while a French survey reported that 72.5% of the physicians (GPs) took further action after a normal PSA result [18]. Also, in a US survey, 29% of the PCPs reported advising yearly screening for prostate cancer, even in patients older than 80 years, with GPs advising this more often than internists/medical subspecialists (55.6% vs 13.3%) [25].

### Repeat testing after moderately abnormal PSA (≥ 4 ng/ml)

Only one database study from the USA described that in 56% of the cases requested by internists an abnormal PSA value was repeated, while 86% were referred [21].

Repeat testing was also described in two European studies, but reported cut-off values differed. One survey study defined PSA ≥ 3 ng/ml as abnormal (15.7% repeat testing by GPs) and the second survey study reported a median cut-off of 5.4 ng/ml (55% repeat testing by GPs) [6,23].

### Reasons for repeating and time frame for follow-up

No reasons for referral or repeat testing after an initially normal PSA value were described in the studies included.

Only one survey described the reasons physicians had for not referring after a primary elevated PSA value; the PSA value was considered too low in 70.5% (all < 10 ng/ml), comorbidity too high in 3.6% and other reasons in 4.2% [23]. No time frame for conducting follow-up was reported.

## Discussion

This is the first systematic review of the literature focusing on follow-up policy after a normal or elevated PSA test by GPs and non-urologic hospitalists. Only 15 studies were published on this topic, most of which were of low quality. Furthermore, a large variety of opinions and policies used by general practitioners and non-urologic hospitalists were described on follow-up after a normal or an abnormal PSA test result. Only four studies researched cut-off values for referral or repeat testing. Reasons for repeating a PSA test after an initial normal result were not described (although two studies described testing PSA yearly). Only one study mentioned the reasons for not referring patients after an abnormal PSA test. A time frame for repeating a test was mentioned in none of the studies. Therefore, the strongest conclusion of this systematic review is that there is a large variety in follow-up after primary PSA testing.

### Selection of studies and quality assessment

We conducted this systematic review according to the proposed guidelines [27]. We believe that our search strategy was adequate, because we used broad search terms and only one additional study was included after checking the reference lists of the included studies. A limitation of this study is the small number of studies that was found and the exclusion of one Japanese study due to language restrictions.

We have arbitrarily chosen a cut-off value of 75% for the definition of high quality studies. We could not apply quality scores in further analyses, due to the wide variety of PSA testing policies and the few data this generated. Therefore, we refrained from pooling the data and performing meta-analyses.

The interpretation of our results may be limited to the differences in study design. Database studies may describe follow-up policies more accurately than survey studies do, as the latter may include socially acceptable answers instead of describing the physicians’ true actions. The number of studies included was too small to uncover this - expected - effect of study design on the results.

### Referral after normal PSA test

After a normal PSA test around 7-10% of the patients are referred to a urologist in all physician groups. Referral after an initially normal PSA value seems to have decreased over time, described by database studies in the USA [14,21] and survey studies in UK [22,23]. A possible explanation for this finding is that patients may be referred for other reasons than the PSA value, for instance because of the presence of therapy resistant LUTS.

### Referral after moderately abnormal PSA test (PSA ≥ 4 ng/ml)

Non-urologic hospitalists appeared to refer more patients after an abnormal PSA test than GPs, but seem to refer about as often as PCPs. Only one study described follow-up by non-urologic hospitalists, but this study also showed quite high referral rates for GPs [22].

More patients were referred in the USA [14,16,19,21,25] than in Spain [24]. These differences between countries may reflect differences in local guidelines. Until 2009 the AUA guideline on PSA testing recommended PSA testing for patients 50 years or older with a life expectancy of 10 years or more [28], while the EAU guideline does not recommend such screening behavior [29].

In the USA, referral rates differed over time (1995–1998) [14,16,19,21], which could not be explained by a change in guidelines, but might be explained by the different designs of these four studies.

In the included survey studies a small group of about 20% of physicians report only referring after a PSA value > 10 ng/ml [15,17,20]. All guidelines advise to follow-up an elevated PSA value, but do not mention how or when follow-up after an abnormal PSA test should be conducted.

Only one study mentioned the reasons for not referring after an abnormal PSA test. The main reason was that the PSA value was considered too low, but comorbidity also played a role. Another reason might be that the interpretation and further management of abnormal test results are strongly affected by the physicians' estimation of pretest disease probability [30]. It is not mentioned if these patients underwent repeat testing. By not conducting follow-up testing physicians seem to imply that an abnormal PSA value holds no consequences for these patients. Maybe these PSA tests could have been omitted, because knowing your PSA value to be elevated, can cause a lot of (unnecessary) distress in patients [31].

### Repeat testing after normal PSA test

Repeat PSA testing after a normal PSA test varied greatly. It seemed to be widely used in the USA (as recommended by the AUA guidelines) as well as in the UK [22,25], and to a lesser extent also in France [18], which is not in accordance with the EAU guidelines. At least two studies (one UK, one USA) reported that physicians agree with screening by an annual PSA test [22,25], which is not in accordance with the European guidelines.

All of the studies included and all of the reported guidelines were published before the recent presentation of the ERSPC and the publication of the results of the Prostate, Lung, Colorectal and Ovarian (PLCO) Cancer Screening Trial. Before the publication of the ERSPC and PLCO results there was no clear evidence on the effect of PSA testing. This resulted in different opinions and consecutively different guidelines on PSA testing, not only between but also within countries. The ERSPC showed that PSA-based screening may reduce prostate cancer mortality by 20%, but it remained unclear which patients may benefit from screening [3]. However, the PLCO trial did not show a mortality reduction [32]. Also, a recent meta-analyses on the effect of population based screening showed no significant effect on mortality [33]. We believe that these studies are important to take into account when considering PSA testing in men who request this. Recently, the US Preventive Services Task Force postulated a negative advice on PSA testing [34].

In survey studies, participants may provide desirable answers, which are not in line with their daily practice. We have found no study to support this suggestion.

## Conclusions

Our study shows that follow-up after a normal or raised PSA test by GPs and non-urologic hospitalists varies greatly and seems not to be in accordance with practice guidelines. This could mean suboptimal treatment for some patients and possibly unnecessary distress in others. Further research is necessary to assess the reasons for this follow-up policy.

## Abbreviations

PSA: Prostate specific antigen; LUTS: Lower urinary tract symptoms; BPH: Benign prostate hyperplasia; AUA: American Urological Association; DRE: Digital rectal examination; EAU: European Association of Urology; GPs: General practitioners; PCPs: Primary care practitioners; ERSPC: European Randomized study of Screening for Prostate Cancer; PLCO: Prostate, Lung, Colorectal and Ovarian Cancer Screening Trial.

## Competing interests

The authors declare that they have no competing interests.

## Authors’ contributions

SM carried out the systematic review with SAML. This was supervised by MHH and MHB. Study design and data processing were evaluated by MHH, MHB, RMN, KM and JEHMH. They also helped SM in drafting this manuscript. All authors read and approved the final manuscript.

## Pre-publication history

The pre-publication history for this paper can be accessed here:

http://www.biomedcentral.com/1471-2296/13/100/prepub

## Supplementary Material

Additional file 1 **Appendix 1.** Search terms. **Appendix 2.** Quality score criteria and informativity. Click here for file
