# Supplementary material for: Prostate specific antigen testing policy worldwide varies greatly and seems not to be in accordance with guidelines: a systematic review
Source: BMC Fam Pract. 2012 Oct 11;13:100. doi: 10.1186/1471-2296-13-100 (PMC3528621; doi:10.1186/1471-2296-13-100)
Supplement: Additional file 1 — Appendix 1. Search terms. Appendix 2. Quality score criteria and informativity. [file 1471-2296-13-100-S1.doc]

**Appendix 1 Search terms**

**Keyword Synonym**

PSA [“Prostate-Specific Antigen” [MeSH] OR PSA test*]

GP [“family practice”[MeSH] OR general practitioner* OR general physician* OR family practitioner* OR family physician* OR primary care*]

Non-urologic hospitalist [hospitalist* OR attending physician* OR medical staff OR specialis* OR hospital registrar* OR medical special* OR anesthesiol* OR anaesthesiol* OR cardiol* OR surgeon* OR surgical* OR dermatol* OR gastroenterol* OR gynaecol* OR internal medi* OR pediat* OR paediat* OR neurolog* OR ophthalmol* OR orthopedi* OR psychiat* OR radiolog* OR radioth* OR reumatol* OR dialys*]

Follow-up [follow-up OR “follow up” [MeSH]]

Referral [referral] ^i^

Consultation [consultation] ^i^

^i^ Consultation and referral not used as MeSH terms, because of too little search results.

**Appendix 2 Quality score criteria and informativity**

**External validity**

*Selection of the study population*

A. Clear description of the research population? ^i^

B. Inclusion en exclusion criteria described?

*Participants and non-responders*

C. Response rate > 70%, or sufficient information on non-responders? ^ii^

*Relationship with source population*

D. Extrapolating results possible for the complete population?

*Description of the study period*

E. Clear description of the study period?

**Internal validity**

*Data collection*

F. Data prospectively collected?

*Measurement instrument*

G. Measuring instrument validated? ^iii^

H. Measuring period clearly described? ^iv^

I. Studied conditions clearly defined?

*Confounders*

J. Confounders described?

**Informativity** ^v^

K. Clear theoretical introduction with relevant references to support the research question?

L. Aims of the study clearly described?

M. Research questions being answered?

N. Clear description of the way data were analysed?

O. Enough original data to evaluate their interpretation?

^i^ Two or more of the following: age distribution, relevant comorbidity, family affected, socio-economic status (SES). For physicians: sex, age, working experience, specialism, high, mean or low SES practice.

ii / iii / iv Not suited for quality measurement of database studies.

^v^ Informativity was not included in the quality score. It was used to assess the usefulness of the study for this systematic review.
